# The Food Equity and Environmental Data Sovereignty (FEEDS) Project: Protocol for a Quasi-Experimental Study Evaluating a Digital Platform for Climate Change Preparedness

**DOI:** 10.2196/31389

**Published:** 2021-09-15

**Authors:** Jasmin Bhawra, Kelly Skinner, Duane Favel, Brenda Green, Ken Coates, Tarun Reddy Katapally

**Affiliations:** 1 Johnson Shoyama Graduate School of Public Policy University of Saskatchewan Saskatoon, SK Canada; 2 School of Public Health Sciences University of Waterloo Waterloo, ON Canada; 3 Northern Village of Île-à-la-Crosse Île-à-la-Crosse, SK Canada; 4 Île-à-la-Crosse School Division Île-à-la-Crosse, SK Canada; 5 Johnson Shoyama Graduate School of Public Policy University of Regina Regina, SK Canada

**Keywords:** food security, food sovereignty, food equity, mental health, solastalgia, climate change impacts, climate change preparedness, digital health, digital dashboards, Indigenous health, mobile phone

## Abstract

**Background:**

Despite having the tools at our disposal to enable an adequate food supply for all people, inequities in food acquisition, distribution, and most importantly, food sovereignty, worsen food insecurity. The detrimental impact of climate change on food systems and mental health is further exacerbated by a lack of food sovereignty. We urgently require innovative solutions to enable food sovereignty, minimize food insecurity, and address climate change–related mental distress (ie, solastalgia). Indigenous communities have a wealth of Traditional Knowledge for climate change adaptation and preparedness to strengthen food systems. Traditional Knowledge combined with Western methods can revolutionize ethical data collection, engagement, and knowledge mobilization.

**Objective:**

The Food Equity and Environmental Data Sovereignty (FEEDS) Project takes a participatory action, citizen science approach for early detection and warning of climate change impacts on food sovereignty, food security, and solastalgia. The aim of this project is to develop and implement a sustainable digital platform that enables real-time decision-making to mitigate climate change–related impacts on food systems and mental well-being.

**Methods:**

Citizen science enables citizens to actively contribute to all aspects of the research process. The FEEDS Project is being implemented in five phases: participatory project planning, digital climate change platform customization, community-led evaluation, digital platform and project refinement, and integrated knowledge translation. The project is governed by a Citizen Scientist Advisory Council comprising Elders, Traditional Knowledge Keepers, key community decision makers, youth, and FEEDS Project researchers. The Council governs all phases of the project, including coconceptualizing a climate change platform, which consists of a smartphone app and a digital decision-making dashboard. Apart from capturing environmental and health-related big data (eg, weather, permafrost degradation, fire hazards, and human movement), the custom-built app uses artificial intelligence to engage and enable citizens to report on environmental hazards, changes in biodiversity or wildlife, and related food and mental health issues in their communities. The app provides citizens with valuable information to mitigate health-related risks and relays big data in real time to a digital dashboard.

**Results:**

This project is currently in phase 1, with the subarctic Métis jurisdiction of Île-à-la-Crosse, Saskatchewan, Canada.

**Conclusions:**

The FEEDS Project facilitates Indigenous Peoples’ self-determination, governance, and data sovereignty. All citizen data are anonymous and encrypted, and communities have ownership, access, control, and possession of their data. The digital dashboard system provides decision makers with real-time data, thereby increasing the capacity to self-govern. The participatory action research approach, combined with digital citizen science, advances the cocreation of knowledge and multidisciplinary collaboration in the digital age. Given the urgency of climate change, leveraging technology provides communities with tools to respond to existing and emerging crises in a timely manner, as well as scientific evidence regarding the urgency of current health and environmental issues.

**International Registered Report Identifier (IRRID):**

PRR1-10.2196/31389

## Introduction

### Background

Climate change poses an existential threat, with inevitable consequences for human health and food systems [1-6]. The global impact of climate change is well established and is commonly classified into direct and indirect effects, ranging from extreme heat and poor air quality to extreme weather events that damage infrastructure and increase health risks [7-9]. The complex and nuanced effects of climate change on food security and sovereignty warrant special attention as we strive for inclusive and equitable climate action.

Food systems include interlinked systems of production, processing, distribution, and consumption [5,10,11]. Indigenous food systems are particularly sensitive to climate change, as many Indigenous communities live in areas experiencing rapid environmental changes. Moreover, competing demands for land due to expanding resource extraction have adversely impacted the subsistence of Indigenous communities [12-16]. As a result, even subtle changes in the environment can have a disproportionately greater impact on Indigenous Peoples’ food security and sovereignty [17-19].

Food security exists “when all people, at all times, have physical, social, and economic access to sufficient, safe, and nutritious food which meets their dietary needs and food preferences for a healthy, active life” [20]. For many Indigenous communities, the issue of food sovereignty is as critical to their survival as having adequate food supply [21-26]. Food sovereignty refers to the “right of local peoples to control their own food systems, including markets, ecological resources, food cultures, and production modes” [27]. This includes the right to define their own food systems, the ability to make decisions about consumption, harvesting practices, and relationship with the land [24,25,28-30]. Food sovereignty is a necessary component of cultural food security—a distinct but overlapping concept [24]. Households or communities can have food security without food sovereignty; however, there is no sovereignty without food security [21-24,31].

Despite having the tools at our disposal to enable adequate food supply for all, inequities in income, food access and distribution, and most importantly, food sovereignty, worsen food insecurity [30,32-34]. The detrimental impact of climate change on food systems is further exacerbated by a lack of food sovereignty [23,35-38]. Failure of these systems is linked to a troubling decline in mental health [39,40] and diet-related chronic disease (ie, type 2 diabetes, obesity, and hypertension) [41,42], particularly among Indigenous and racialized communities. These communities also face the brunt of climate change impacts and have the least control over their food access [13,43-48]. Solastalgia, which refers to the mental distress caused by environmental degradation and changes, is of increasing concern as environmental disasters and biodiversity loss have become more frequent and severe due to climate change [49]. Solastalgia may include ecological grief or eco-anxiety resulting from unanticipated ecological losses and uncertainty about the changing environment [49].

In rural and remote communities, climate change–related risks and their interplay with food systems and solastalgia can be consistently monitored using sophisticated early warning and response systems to improve the management of food and related mental health crises [1,50,51]. Such advanced monitoring can be facilitated through real-time engagement via ubiquitous digital devices such as smartphones. Smartphones can serve as tools of equity by amplifying the voices of isolated citizens and communities, as well as providing citizens with timely access to resources and information [52-54]. Community-based data collection improves monitoring and adaptation to environmental changes as Indigenous communities have a wealth of knowledge and experience in coping with environmental changes [1,55-57]. The integration of Western methods and Traditional Knowledge using Two-Eyed Seeing approaches is critical for developing and implementing climate change solutions in partnership with Indigenous communities [1,58-62].

### Objective

We urgently require innovative solutions to enable food sovereignty and minimize food insecurity and solastalgia. In the digital age, citizen science can revolutionize ethical data collection, engagement, and knowledge mobilization. The Food Equity and Environmental Data Sovereignty (FEEDS) Project takes a participatory action, citizen science approach for early detection and warning of climate change impacts on food security, sovereignty, and solastalgia. The objective of this project is to customize and implement a sustainable digital platform that enables real-time decision-making and engagement among community members, decision makers, and researchers. On the basis of the principles of Two-Eyed Seeing [60], this study aims to partner with Indigenous communities for early detection and management of climate change as it relates to probable impacts on food systems and human health using a digital rapid response platform. This paper describes the FEEDS Project protocol and its application in a subarctic Indigenous community in Canada.

## Methods

### Study Design

The FEEDS study is being implemented using a quasi-experimental design, which enables exploration of the links between environment, mental health, and food systems. The primary goal is to understand how early detection of climate change risks influences decision-making to improve management of solastalgia, food access and acquisition practices. The quasi-experimental design allows for the capture of natural experiments (ie, food system impacts resulting from environmental changes such as early ice road thaw) and pre- and posttests to assess the influence of specific climate change adaptation strategies. Pre- and posttests will include collecting data at baseline (before the implementation of a climate change preparedness or adaptation strategy) and at several time points after the implementation of a strategy. These data will elucidate changes in the knowledge or perceptions of community members about climate change impacts, changes in preparedness and adaptation behaviors, and specific effects of strategies on outcomes including food security status, food sovereignty, and solastalgia. Overall, this study design provides the flexibility required to adapt to emerging community needs over time.

FEEDS is part of the Smart Platform (DEPtH Lab) [53], a digital epidemiological and citizen science initiative that enables ethical surveillance, integrated knowledge translation, and behavioral and policy interventions. This platform is informed by the Smart Framework, a theoretical framework [52] that integrates citizen science, community-based participatory research, and systems science to conduct population health research in the digital age.

Citizen science facilitates active citizen participation in all phases of research, and when combined with community-based research methods, it can enable local solutions to global problems [52]. When added to systems science, this approach offers a unique opportunity to capture a holistic perspective and unpack underlying mechanisms for complex problems using big data [52]. More importantly, in partnership with Indigenous researchers, the platform integrates the Smart Framework with the principles of Ownership, Control, Access, and Possession [63] to not only coconceptualize features and cocreate knowledge but also to ensure data sovereignty through community ownership of data. The FEEDS study highlights Traditional Knowledge about the environment and food systems, Indigenous research methods, and Western digital citizen science methods to facilitate Two-Eyed Seeing.

### Setting

This project is being implemented first in a subarctic community with road access in northwest Saskatchewan, Canada, the Northern Village of Île-à-la-Crosse. Sakitawak, the Cree name for Île-à-la-Crosse, translates to *the place where the river flows out* [64]. Sakitawak represents the geographic location of the community on the lake of Île-à-la-Crosse, which made it a strategic location for the fur trade. Established in 1778, Île-à-la-Crosse is the second oldest community in Saskatchewan, with a population of approximately 1300 [64]. The median age of residents was 29 years in 2016, with approximately 65% of residents aged 15 to 64 years. Île-à-la-Crosse is a predominantly Métis community (77%), and Northern Michif is the traditional language [65]. The community celebrates the heritages of both European and First Nations. Commercial fishing, wild rice harvesting, forestry, a hospital, and a school are the primary sources of employment in the community. Mobile and Wi-Fi internet plans provide data access to community members, with most citizens aged ≥13 years owning smartphones. Moreover, the presence of a cell tower in Île-à-la-Crosse offers reliable access to cellular data. Wi-Fi and cellular data access vary widely within and between communities; hence, it is not always the case that remote communities have lower access.

### Citizen Scientist Recruitment and Engagement

Citizen science can range from contribution and collaboration to the cocreation of knowledge with citizens [52]. This project was coconceptualized with the Île-à-la-Crosse Citizen Scientist Advisory Council, which includes youth, Elders, Indigenous Knowledge Keepers, and decision makers of the jurisdiction of Île-à-la-Crosse. The primary role of the Advisory Council is to represent the interests of the community members and guide the governance of project development, implementation, and evaluation. All Council members are provided with Can $150 (US $119.30) as honoraria for each meeting to respect their time and guidance.

The Council leads the citizen recruitment strategy. Community members aged 13 years and older are invited to participate in the project as citizen scientists. Citizen scientists can actively engage in the research process from data collection to knowledge translation [52,53,66]. Citizen scientists determine their level of participation, but unlike traditional research projects, they can contribute to cocreating project objectives as community needs change. This participatory approach ensures that citizens are not passively providing data and can instead engage with researchers and decision makers to shape solutions or outcomes of interest. For example, citizens can anonymously and directly communicate information about a community emergency with decision makers through a front-end mobile app via a user-triggered messaging system. Citizen science also plays an important role in facilitating self-determination and self-governance, as citizens are stewards of their own data and decision-making in the community.

To obtain a representative community sample, the recruitment and engagement strategy aims to enroll citizens across various sociodemographic, gender, and digital literacy categories. Key decision makers and knowledge keepers are first approached to identify appropriate venues for engagement to invite citizens to the FEEDS Project. Inclusion criteria includes citizens aged ≥13 years who own or have access to smartphones. Citizens are recruited by disseminating project information through social media, the community radio station, the office of the Mayor, and the email list of the school board. Given the urgent and sensitive nature of addressing climate change impacts on mental health and food systems, the Advisory Council does not consider it ethical to randomize or limit citizen participation in this project [67].

### Study Protocol

#### Overview

**Figure 1:**
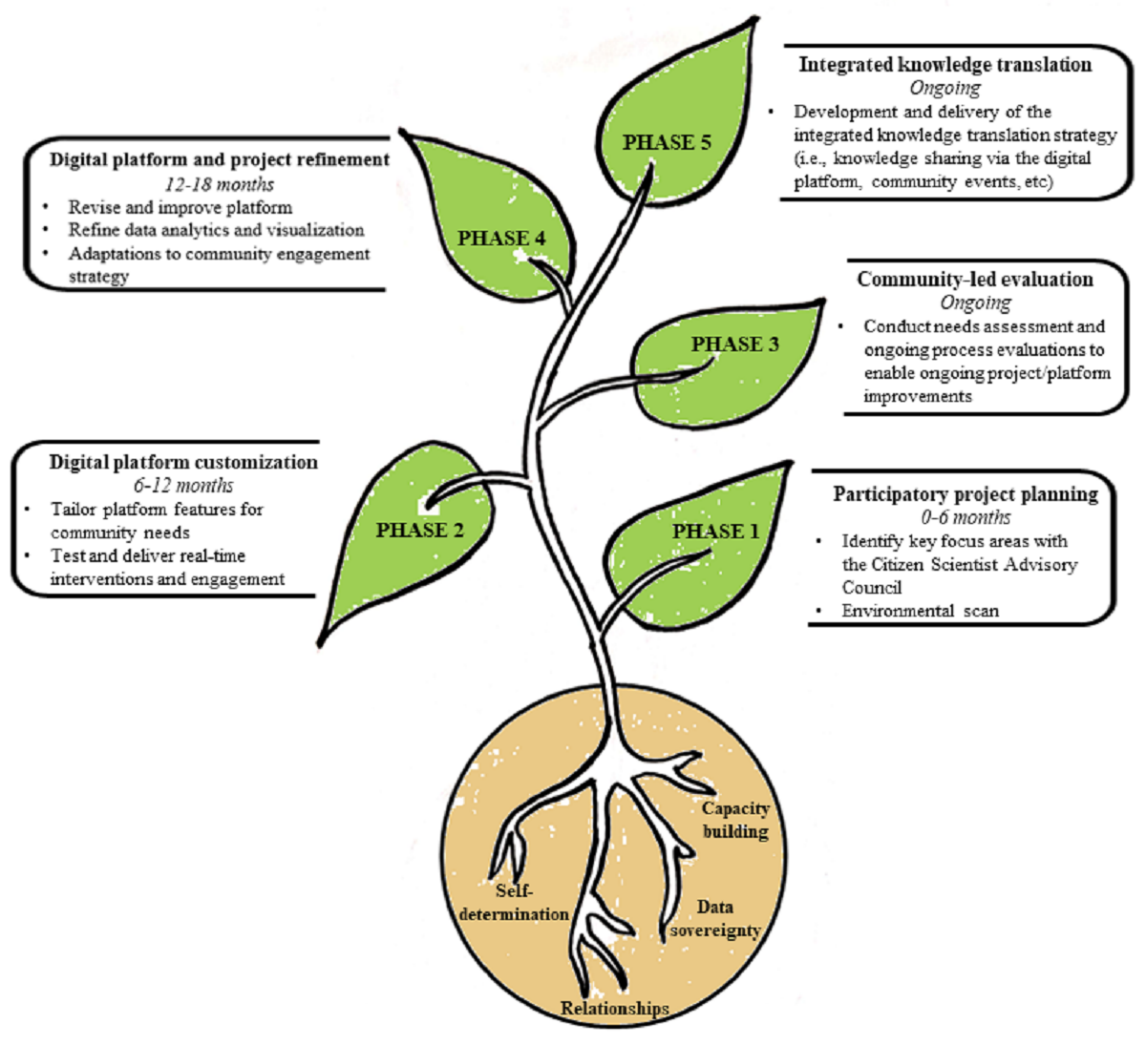
The Food Equity and Environmental Data Sovereignty Project overview.

This study is being implemented in five iterative phases in partnership with the Northern Village of Île-à-la-Crosse. The phases include: (1) participatory project planning, (2) digital platform customization, (3) community-led evaluation, (4) digital platform and project refinement, and (5) integrated knowledge translation. Figure 1 summarizes the FEEDS Project phases (leaves), associated tasks and timelines, and the core principles (roots) of self-determination, relationships with communities, data sovereignty, and capacity building.

#### Phase 1: Participatory Project Planning (0 to 6 Months)

The project is currently in phase 1, with the Advisory Council convening with the lead FEEDS researchers once every 2 months. The main agenda for the Council meetings revolves around the following themes: (1) rapid response to environmental disasters or hazards; (2) sustainability of the digital platform; (3) food and data sovereignty; (4) real-time intervention design for mental wellness; (5) citizen engagement features of the smartphone app; and (6) decision-making dashboard features and functionality. As part of this process, the Council has identified existing and emerging needs to address climate change–related impacts on community health. In phase 1, we are completing an environmental scan to create an inventory of available resources and programming for food security and sovereignty, solastalgia, and mental wellness, as well as climate change adaptation and preparedness strategies in the community. The findings from this scan will inform digital climate change platform customization.

#### Phase 2: Digital Climate Change Platform Customization (6-12 Months)

As part of the Smart Platform [53], a digital platform has been developed to address existing and emerging population health crises. Using citizen-owned smartphones, a mobile app can be downloaded by citizens to share information about relevant community issues. Data from this app are relayed in real time to a digital decision-making dashboard that is securely accessed by the Mayor of Île-à-la-Crosse.

**Figure 2:**
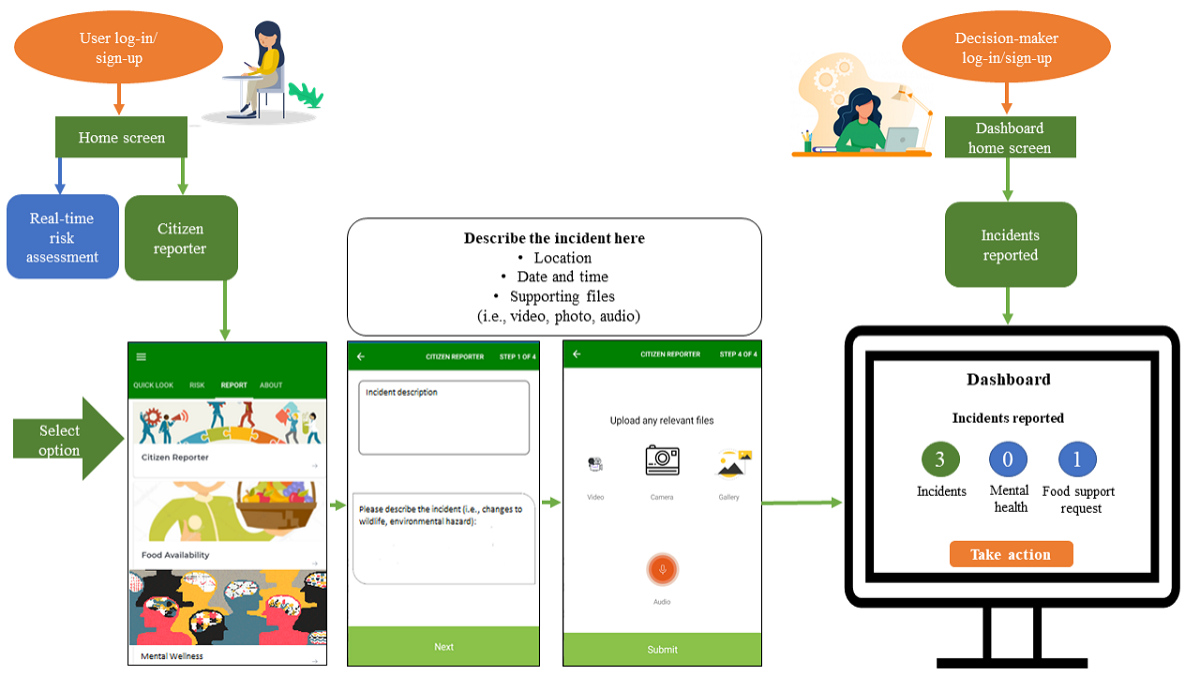
Prototype of the digital dashboard for decision-making.

Given the increasing concern over the adverse impacts of climate change on food systems and health, this digital platform is being adapted to capture relevant environmental, food, and solastalgia-related data in the community of Île-à-la-Crosse (Figure 2). The Advisory Council is leading the conversation regarding customization of digital platform features to address three key objectives: (1) ethical monitoring of risk that is conducted by engaging citizens in real time, (2) real-time interventions to mitigate risk by providing citizens with community-specific alerts, and (3) implementation of a digital decision-making dashboard to facilitate Indigenous self-governance and data sovereignty. There are three guiding principles that will enable the achievement of these objectives: (1) citizen empowerment and data ownership to maximize active engagement, (2) privacy to ensure that sensitive data such as citizen location are not stored in external servers [52,53], and (3) security and scalability to replicate the platform across multiple jurisdictions. The office of the Mayor is leading the development and coordination of rapid response strategies in the community.

#### Phase 3: Community-Led Evaluation (Ongoing)

After an initial needs assessment, process evaluations will be conducted throughout the project to ensure that the app and dashboard are designed with relevant features for citizens and decision makers. Textbox 1 summarizes the key questions to be addressed by each evaluation type. The timeline of the evaluations is set by the Advisory Council; however, a needs assessment was conducted at the beginning of the project, with process evaluations scheduled annually. A consistent evaluation will ensure that updates and improvements are made to the platform in a timely manner.

**Summary of evaluation activities.**

**Needs assessment (year 1)**

- What are the key concerns of the community regarding?
  Food security and sovereignty
  Climate change and environmental events or hazards
  Existing and emerging mental health issues as they relate to climate change (ie, solastalgia and eco-anxiety)
- What types of programming, services, or resources are available to address?
  Food insecurity and sovereignty (including but not limited to food access, availability, and traditional food acquisition practices)
  Climate change and/or environmental events (ie, environmental hazards and changes in biodiversity and/or wildlife)
  Community mental health
- What technology, if any, is the community currently using to address concerns above?
- What services, resources, and knowledge would help improve the community’s response to concerns above?
- How does the community envision the digital platform (app+dashboard) to address climate change, food systems, and related mental health issues?
- What features would be useful in the digital platform?

**Engagement or evaluation activity**

- Environmental scan
- Advisory Council consultation
- Key informant interviews
  Indigenous Knowledge Keepers
  Community Mayor

**Process evaluation (annually)**

- To what extent has the digital platform helped the community address issues related to?
  Food security and sovereignty
  Climate change and environmental events or hazards
  Related mental health issues
- To what extent has the digital dashboard helped decision makers respond to issues related to the concerns above?
- What are some areas of improvement for the digital platform (app+dashboard)?
- Are there differences between app users and nonusers?
- Are certain subgroups more or less likely to use certain app features?

**Engagement or evaluation activity**

- Key informant interviews
- Evaluation survey administered via smartphone app (modified Mobile App Rating Scale [68] and Knowledge Uptake and Utilization Tool [69])
- Focus groups with citizens (groups of 6-8 people with representation from sex- and gender-diverse citizens, across age groups, as well as app user and nonuser groups)

#### Phase 4: Digital Platform and Project Refinement (12-18 Months)

Consistent, evidence-based adaptation of the digital platform will be conducted using big data ethically sourced from citizens. These adaptations ensure that both dashboard data analytics and app-based engagement are continuously enhanced. As digital dashboards are designed in close collaboration with the decision makers, the evaluation activities will engage decision makers at regular intervals to obtain their feedback to advance data analytics based on their needs. This process is also part of the overarching phase of integrated knowledge translation.

Real-time engagement is a critical part of this project; thus, the engagement strategy will be adapted and refined throughout the project to encourage citizen participation. Using human-centered artificial intelligence and citizen science, the platform improves engagement continuously by refining the app based on citizen input. As engagement will depend on behaviors, emotions, expectations, and perceptions of the citizens, human-centered artificial intelligence will develop solutions that will capture the context and intention of these factors to further human-machine (app) interactions. For example, if a particular citizen is engaging more during the early morning due to their work schedule, the platform will develop algorithms to engage that citizen in the morning. This approach will enhance evidence-based engagement, improve the reliability and validity of artificial intelligence algorithms, and continuously refine the product codeveloped with citizens.

#### Overarching Phase: Integrated Knowledge Translation (Ongoing)

The success of this platform will depend on the overarching phase of integrated knowledge translation that integrates the Smart Framework [52] with Traditional Knowledge to ensure Two-Eyed Seeing [60] in the project conceptualization, development, implementation, evaluation, and refinement. The Smart Platform combines citizen science, community-based participatory research, and systems science to conduct population health intervention research in the digital age [52,53]. By integrating this approach of the framework with the Traditional Knowledge of Indigenous communities, we are working to decolonize how big data and artificial intelligence are used in advancing equity. Integrated knowledge translation ultimately promotes community input for climate adaptation strategies at every phase and is critical for vertical (to larger communities) and horizontal (increasing platform features) scale-up.

Knowledge translation activities include knowledge sharing via the digital platform with citizens and decision makers, web-based and in-person events, social media, local radio, as well as reports and publications. An important aspect of data sovereignty is the control of where, with whom, how, and when project data are shared. Hence, the data collected through the FEEDS Project belong to the citizens and community where the project is based, and Île-à-la-Crosse along with the Citizen Scientist Advisory Council guides data governance.

### Data Collection and Analysis

This project collects quantitative and qualitative data by engaging citizens through the FEEDS app. In particular, data on environmental changes, hazards, and events are collected to track the environmental impacts on community food systems and mental health. Food-specific data, including food availability, access, acquisition practices, and connections to environmental conditions, are used to identify resources, programming, or policy solutions within the community. Impacts on health are also tracked by asking citizens about their mental, physical, spiritual, and emotional well-being. These time-stamped big data include both subjective ecological momentary assessments and objective sensor data [52].

Traditional Knowledge, citizen perspectives, and decision maker feedback are critical to understanding knowledge uptake and use, as well as designing and delivering culturally responsive interventions for climate change preparedness, food sovereignty, and solastalgia. The smartphone app, focus group discussions, and key informant interviews will capture relevant contextual information to understand specific issues in the community, barriers to, and opportunities for climate change adaptation and preparedness. Qualitative data collection, particularly digital storytelling, can play an important role in passing Traditional Knowledge to future generations in the community.

Big data from FEEDS can be linked with data from other databases, including repositories of climate change (ie, Arctic observatories) and weather data (ie, Environment and Climate Change Canada) to leverage existing sources that have historical and prospective data that will enhance prediction models.

All citizen data will be anonymized; however, citizens have the option to identify themselves to community decision makers to seek help. Before data collection, citizens provide informed consent using the app. To ensure confidentiality, data are encrypted before being stored on smartphones and streamed to servers when the devices establish a Wi-Fi connection. Permissions built into the app are restricted so that the app cannot access personally identifiable information that is present on smartphones (eg, contact lists or network sites visited). MAC address anonymization is used to protect citizen scientists’ data. Citizens are also introduced to the *pause* functionality—a key privacy component—which allows participants to disable monitoring for a set duration. In the case of lost or stolen phones, participants do not have to worry about data breaches, as study data on phones are strongly encrypted. Once data have been uploaded, they are stored on the secure University of Saskatchewan servers for 10 years.

All citizen scientists will have the option to drop out of the study through the app anytime they wish. Moreover, citizens will be provided with study emails to contact the investigators with questions and concerns and to remove themselves from the study anytime they wish.

Mixed methods analyses will be conducted immediately after data collection to ensure timely dissemination of findings, which may impact app features and/or community decision-making. Each short- and long-term objective will be assessed beginning with descriptive analyses to generate accurate population or subpopulation profiles. This will enable all further regression analyses to consider the intersection of multiple identities and sex and gender variations. Profiles that reflect the behavior or outcomes of subpopulations will be constructed. For example, sex and gender differences in the perception of app features and usefulness in association with age and digital literacy status (urban, rural, and remote) will be delineated to enable refinement of the platform. Missing data will be rigorously explored and addressed appropriately to ensure maximum use of objective and subjective data. Missing patterns will be explored before applying imputation or deletion strategies for different mechanisms: ignorable missing (random missing) and nonignorable missing [70]. Comprehensive exploration will also be conducted to identify potential ignorable missing (random) or nonignorable missing data across the intersection of sex and gender spectra. As variation in app use is expected among citizens, understanding and addressing missing data through imputation or deletion strategies will be critical to address the project objectives [70].

## Results

The FEEDS Project is currently in phase 1. The Advisory Council is comprised of 8 citizen scientists, including 4 youth, 2 Elders, and 2 decision makers. Other communities in the north will be invited to collaborate and customize the digital platform as part of the FEEDS scale-up. The FEEDS Project was approved by the research ethics boards of the University of Regina and the University of Saskatchewan through a synchronized review protocol (REB# 2017-29).

## Discussion

Food equity and environmental data sovereignty are key components of climate justice. Food equity includes the right for all people to consume, grow, and acquire healthy and affordable food and involves eliminating systemic barriers within food systems [27,28,71,72]. Food equity requires food security and food sovereignty and promotes the ability of citizens to participate in decision-making about food systems [73,74]. The sovereignty of environmental and health data within this study is critical, especially for Indigenous communities to enable self-governance.

The FEEDS Project takes a decolonized approach to research using the Smart Framework and principles of Two-Eyed Seeing. The unique digital platform promotes data sovereignty, as communities are the stewards of their own data. The issues of climate change, food inequity, and solastalgia require innovative solutions. The rapid response enabled via digital platforms combined with the wealth of Traditional Knowledge about the environment and food systems is essential not only to address these issues in partnership with Indigenous communities but also for conversations about climate action and justice, globally.

Citizen science has played a vital role in the ecological sciences and has great potential to combat existing and emerging health crises if citizens’ data can be anonymized and effectively shared with citizens and their communities [52]. The FEEDS Project demonstrates how advancing citizen science can enable big data generation to address complex issues that intersect multiple disciplines and sectors.

For this initiative to be successful, capacity building is critical for ongoing work with the jurisdiction of Île-à-la-Crosse. The FEEDS Project provides a digital infrastructure for monitoring, managing, and eventually mitigating adverse climate change–related impacts on local food systems and mental health. Ultimately, community capacity will ensure the sustainability of this infrastructure. The Advisory Council provides a space for community members, including youth and Elders, to meet and learn from one another. In Île-à-la-Crosse, adjacent initiatives, including digital literacy programs, are being set up to ensure accessibility and inclusion of community members across all age groups. The FEEDS Project can be adapted and scaled up to other northern communities facing similar environmental and food system challenges.

In addition to the data ownership and privacy concerns covered in the *Methods* section, another contextual consideration for project success is addressing internet inequity. Internet inequity refers to differential access to the internet based on the wealth of a country (high-, low-, or middle-income), geographic region (urban, rural, or remote), and socioeconomic status, gender, age, or ethnicity of the citizens [52]. Rural and remote communities facing barriers to internet connectivity are arguably the most adversely impacted by climate change and food insecurity, so it is important to consider whom we may be leaving out by going digital. The digital divide is a complex phenomenon; however, countries such as Canada are pledging resources to enable equitable access to rural and remote areas [75-78]. Although it is beyond the scope of this project to address these systemic issues, we are working with policymakers and communities as part of the Smart Platform to improve internet access for citizens. For example, we are providing smartphones and data plans to enable citizen participation and working with local community organizations to provide venues for free Wi-Fi access.

The FEEDS Project takes a novel digital citizen science approach to understand and address climate change, food equity, and solastalgia in the 21st century. Climate change is taking an inevitable toll on food systems, with remote, Indigenous communities disproportionately impacted in Canada. Combining the principles of Two-Eyed Seeing and the Smart Framework, the FEEDS Project provides early detection and potential for mitigation of adverse environmental, food system, and mental health impacts, while enabling data sovereignty and self-governance.

## Abbreviations

FEEDS: Food Equity and Environmental Data Sovereignty
